# Supplementary material for: Copper-surface-mediated synthesis of acetylenic carbon-rich nanofibers for active metal-free photocathodes
Source: Nat Commun. 2018 Mar 19;9:1140. doi: 10.1038/s41467-018-03444-0 (PMC5859183; doi:10.1038/s41467-018-03444-0)
Supplement: Supplementary file 1 — Supplementary Information(DOCX 8122 kb) [file 41467_2018_3444_MOESM1_ESM.docx]

**Supplementary Figures**

**
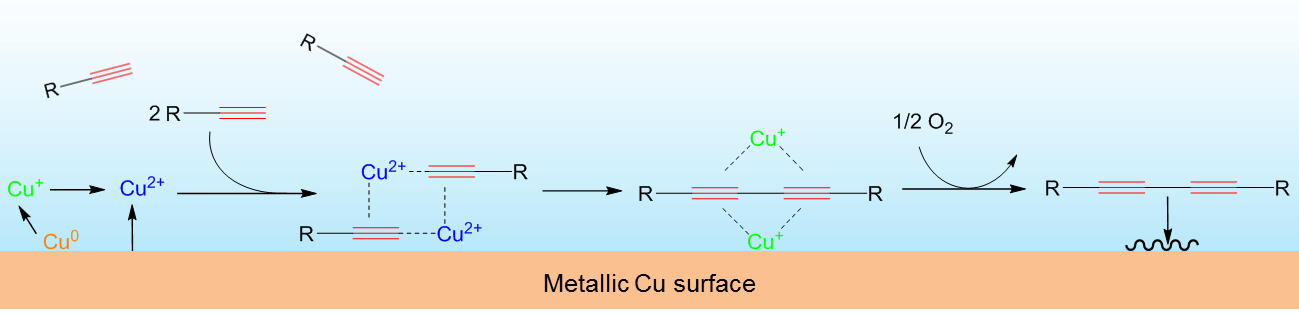
Supplementary Figure 1** | **Schematic illustration the mechanism of Cu-surface mediated Glaser coupling.** The copper catalyzed Glaser coupling reaction takes place at the interface where various copper ions are dissolved, which results in polydiacetylene network uniformly deposited on the copper surface.


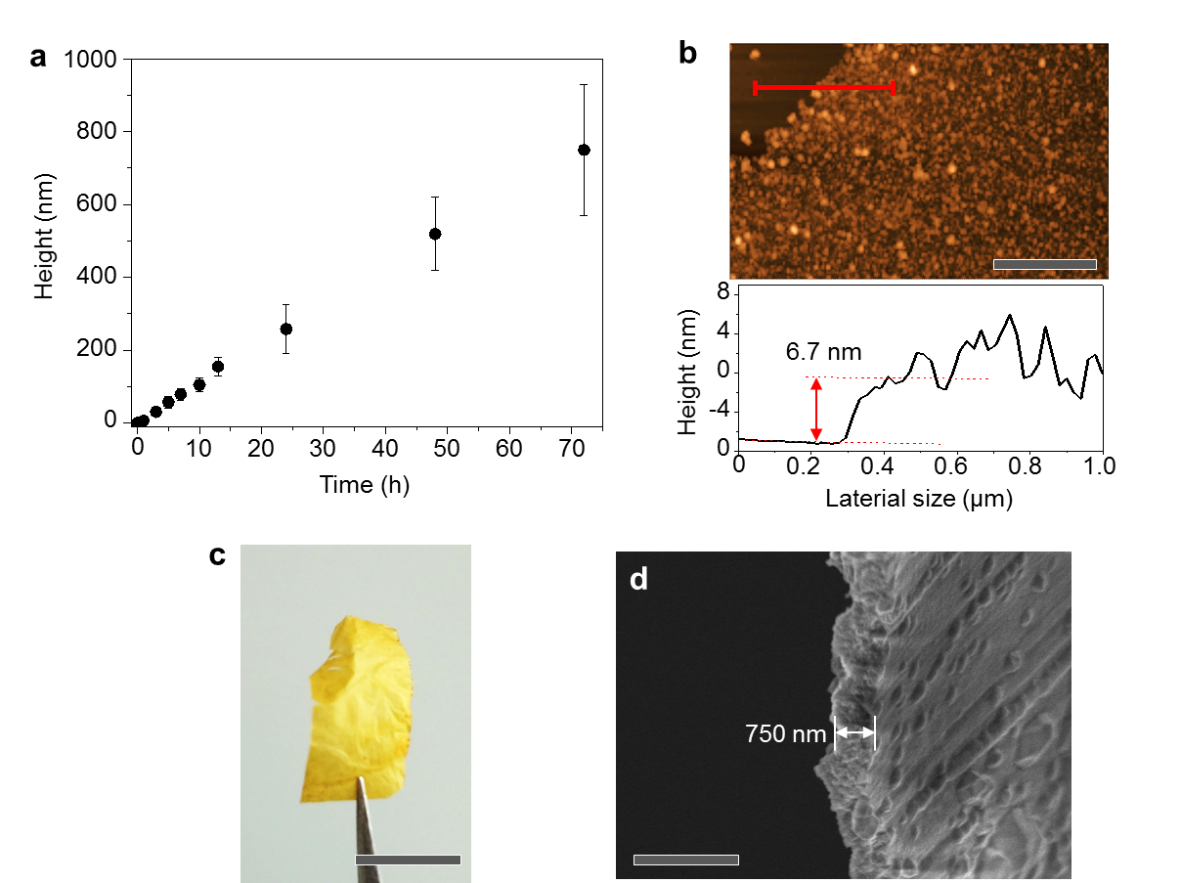


**Supplementary Figure 2** | **Synthesis of PTEB layer with various thickness.** (**a**) Development of the thickness of PTEB nanofiber films with the reaction time. The growth rate (*δ_d_*) of PTEB on copper substrate was determined to be *δ_d_* = 10 nm h^-1^ within the 72 h test. (**b**) AFM topography and height analysis (*h* = 6.7 nm) along the indicated line of the PTEB film prepared in 1 hour on copper surface and transferred onto a SiO_2_ wafer. (**c**) Photograph shows that the PTEB film (in 72 h reaction) can be completely free-standing after dissolving the copper substrate by an aqueous solution of ammonium persulfate (0.25 g mL^-1^). (**d**) The thickness of PTEB film in (**c**) was determined to be *ca.* 750 nm at the SEM cross-section. Scale bars: (**b**) 1 µm; (**c**) 10 mm; (**d**) 2 µm.


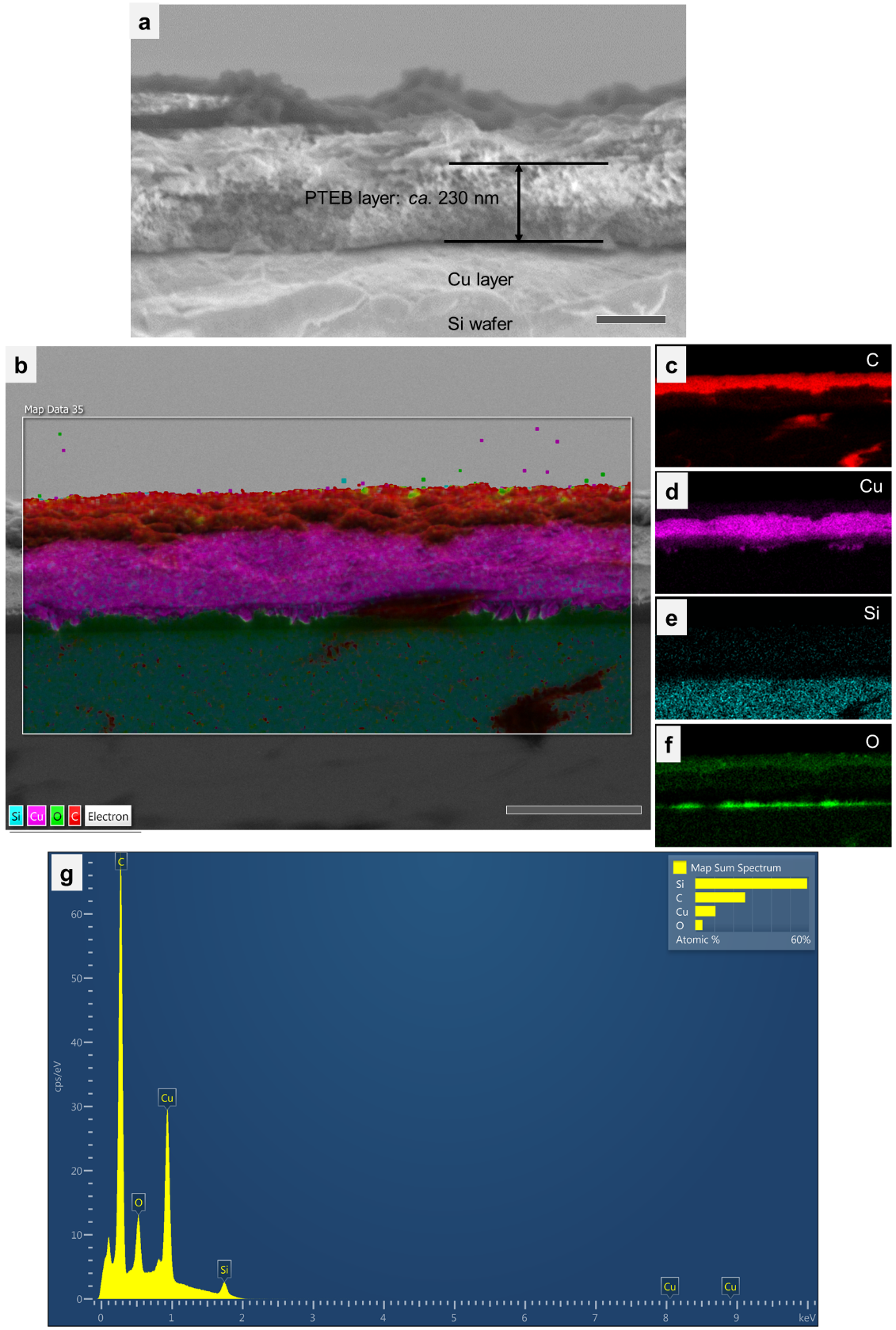


**Supplementary Figure 3** |**Cross-section SEM image and energy dispersive X-ray (EDX) elemental mapping**. A PTEB layer of *ca.* 230 nm thick can be identified in the (**a**) cross-section SEM image of the sample on a copper coated Si wafer. Further EDX elemental mapping images show clear contrast of different layers on the sample: (**b**) full elements mapping, (**c**) carbon, (**d**) copper, (**e**) silicon and (**f**) oxygen. (**g**) The corresponding EDX spectrum of (**b**) measured at 3 kV acceleration voltage. Scale bars: (**a**) 200 nm; (**b**) 2.5 µm.


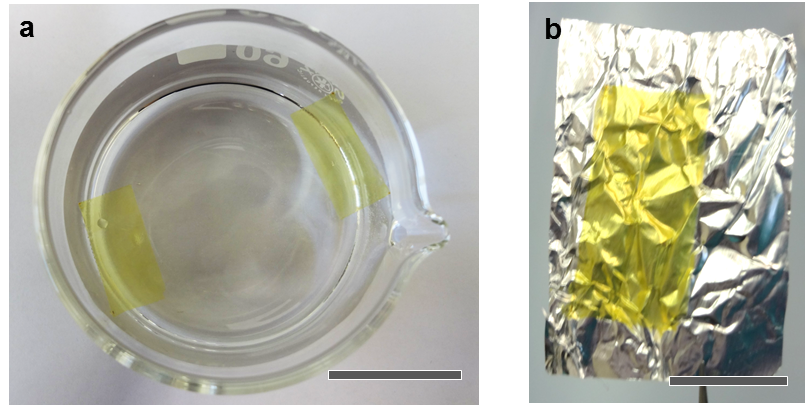


**Supplementary Figure 4** | **Photographs of the transferring process.** (**a**) Free-standing PTEB films floating on the water surface after etching the copper substrate. (**b**) The film can be readily transferred onto any substrate even curved aluminum foil. Scale bars: (**a**) 30 mm; (**b**) 10 mm.


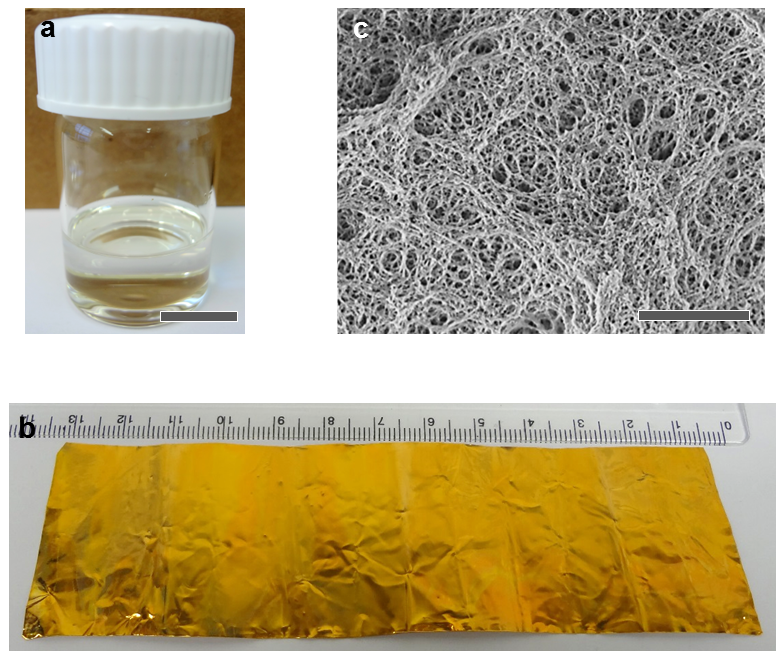


**Supplementary Figure 5** | **Large-area PTEB nanofibers grown on copper foil.** (**a**) Monomer solution: 0.5 g mL^-1^ of 1,3,5-triethynylbenzene (TEB) in 10 mL pyridine. (**b**) Photograph of uniform and large area (*ca.* 4 × 12 cm^2^) PTEB film prepared from the monomer solution shown in a. (**c**) A magnification of (**b**) shows that the film is composed by interconnected nanofibers with a diameter range from *ca.* 10 nm to *ca.* 20 nm. Scale bars: (**a**) 10 mm; (**c**) 500 nm.


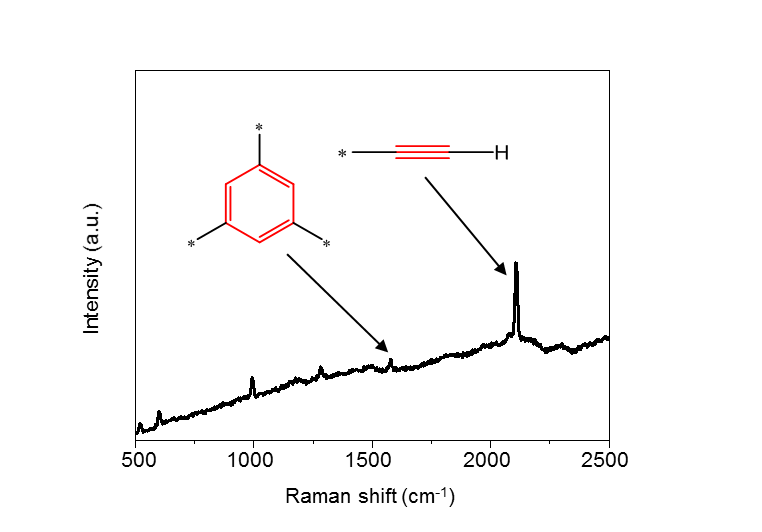


**Supplementary Figure 6** | **Raman spectrum of TEB monomer.** The consumption of the C≡C triple bond can be readily determined by the disappearance of the characteristic terminal alkyne vibrational band at 2106 cm^-1^.


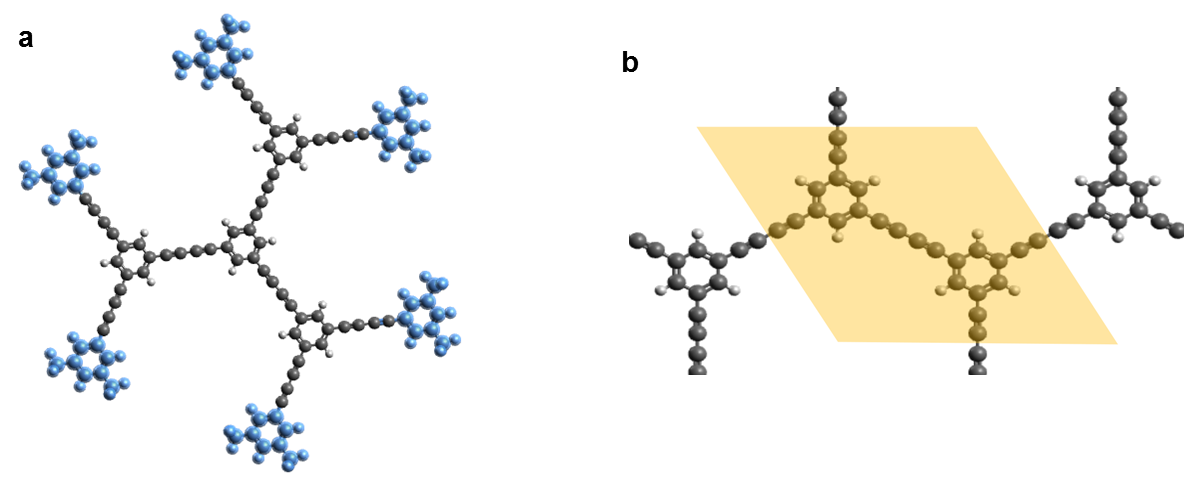


**Supplementary Figure 7** | **DFT calculations of PTEB structure.** (**a**) Structure of the cluster model used for DFT calculations (Gaussian09D) of the Raman response carried out with Gaussian09D. In the simulation of the Raman spectrum (Fig. 1f) the highlighted atoms have been assigned a mass of 100 *amu*, so to limit spurious effects due to the terminal atoms of the finite cluster model. (**b**) Unit cell adopted in the periodic boundary condition calculation of the Raman spectrum (Crystal14, see Supplementary Fig. 8 below).

**
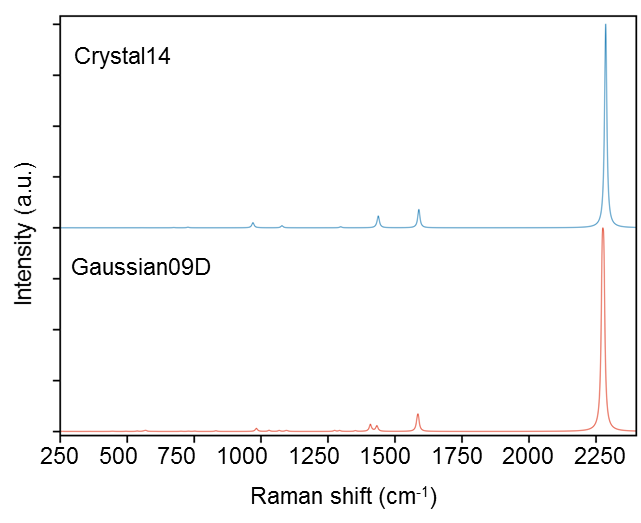
**

**Supplementary Figure 8** | Comparison of calculated Raman response carried out with Gaussian09D and Crystal14.

**
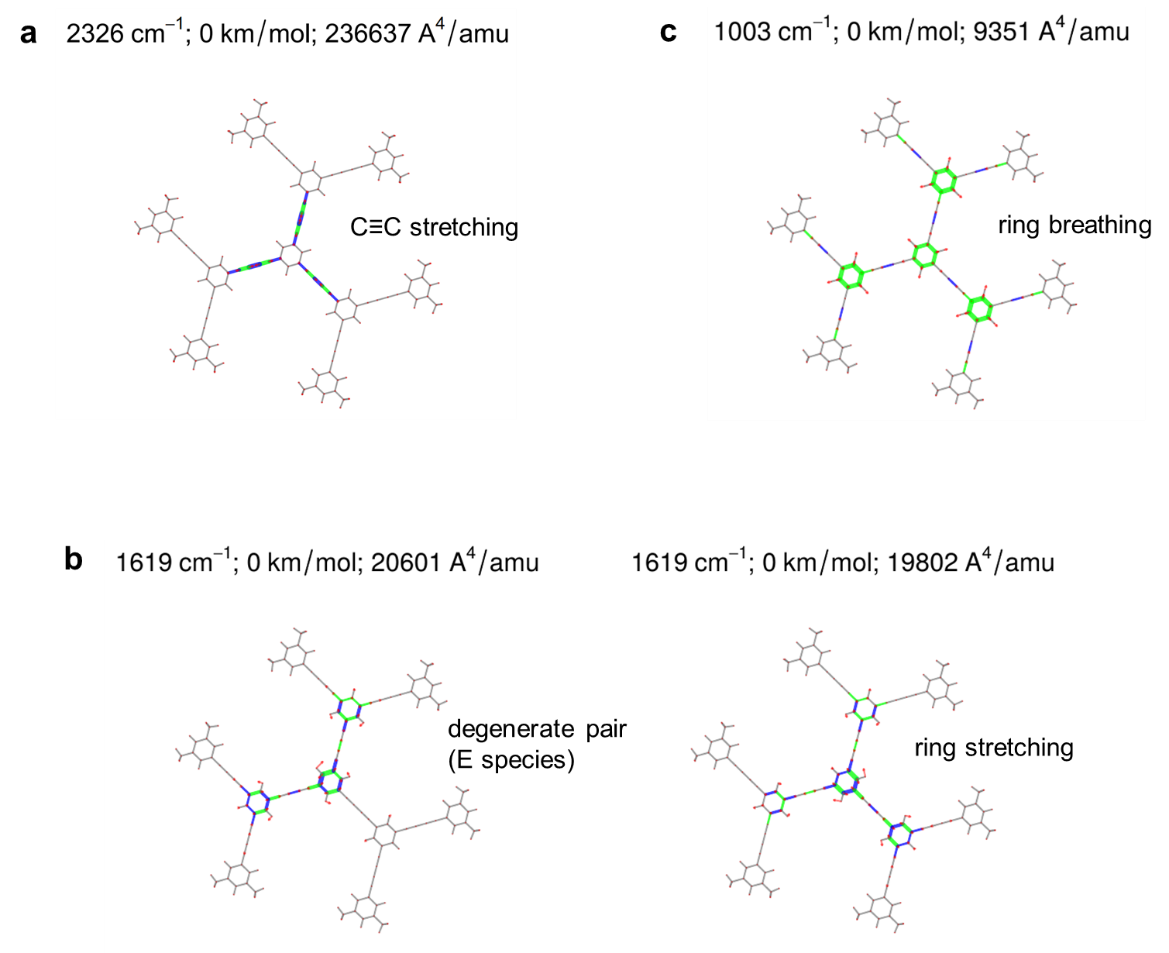
**

**Supplementary Figure 9** | **Sketch of the normal modes of the cluster model**. The structures are associated with the peaks a, b, and c discussed in the main text (Fig 1f). Red lines represent displacement vectors; C≡C bonds are represented as green (blue) lines of different thicknesses according to their relative stretching (shrinking). Mode assignment: (**a**) C≡C stretching; (**b**) ring stretching (degenerate pair); (**c**) ring breathing. For each mode the wavenumber, Raman activity (A^4^/amu) and IR intensity (km mol^-1^) computed by DFT (B3LYP/6-31G(d,p)) are also reported. To ease the comparison in Fig. 1f (main text) of the simulated Raman spectrum *vs.* the experimental we adopt a scale factor of 0.98 for computed Raman wavenumbers, as customary in literature^1^.


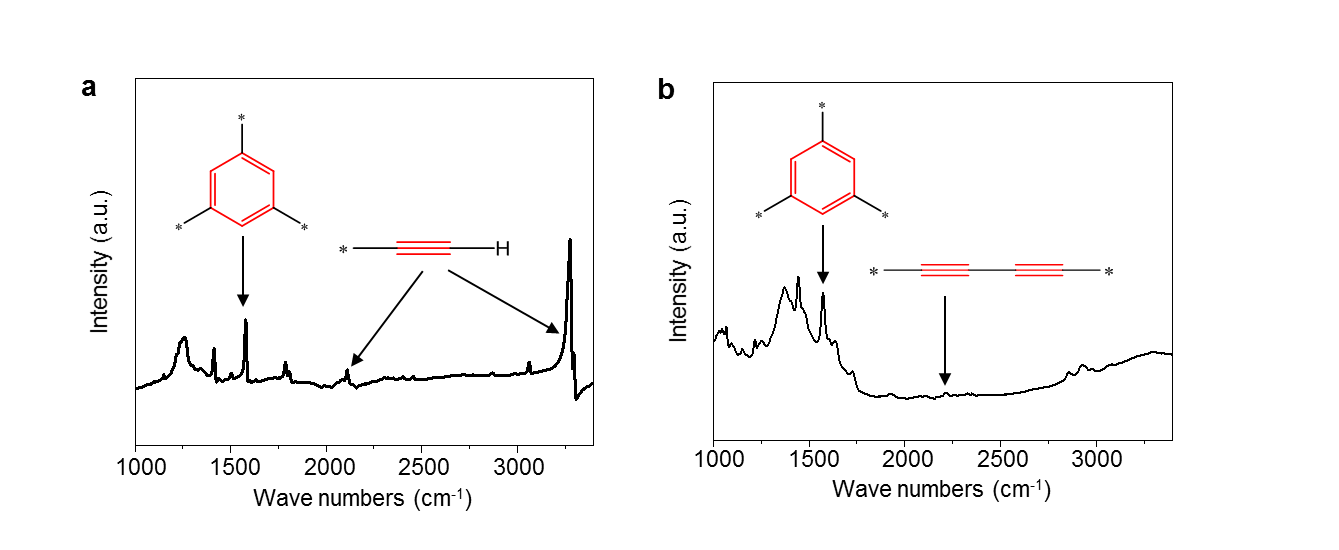


**Supplementary Figure 10** | **Fourier transform infrared spectroscopy (FT-IR) spectrum of TEB and PTEB**. (**a**) TEB monomer. The bands located at 2110 cm^-1^ and 3276 cm^-1^ are the typical terminal C≡C stretching vibration. (**b**) As-grown PTEB nanofibers on copper substrate. The characteristic peaks of terminal C≡C bond disappeared in the PTEB, while the bands at 2215 cm^-1^ arise due to the formation of diacetylenic network. The intensity of which are a slight weak because of the much high conjugation length in the polymer.


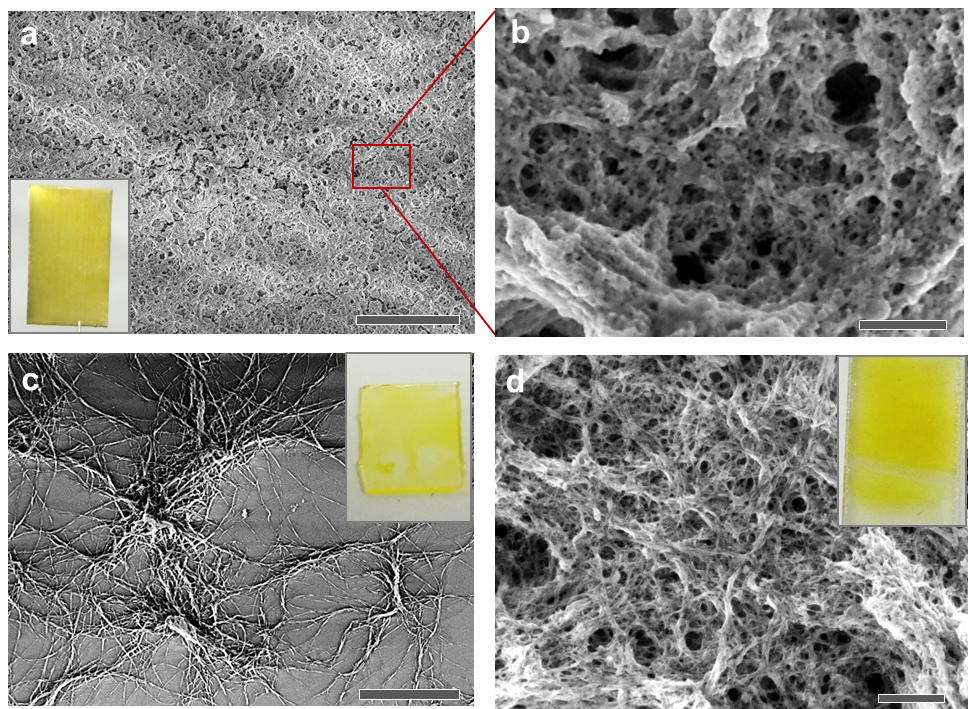


**Supplementary Figure 11** | **Synthesis of PTEB nanofibers on various substrates.** SEM images show that the PTEB nanofibers grown on (**a**) and (**b**) titanium plate, (**c**) glass, (**d**) FTO glass; insets: photographs of each samples. Scale bars: (**a**) 4 µm; (**b**) 100 nm; (**c**) 1 µm; (**d**) 200 nm.

**
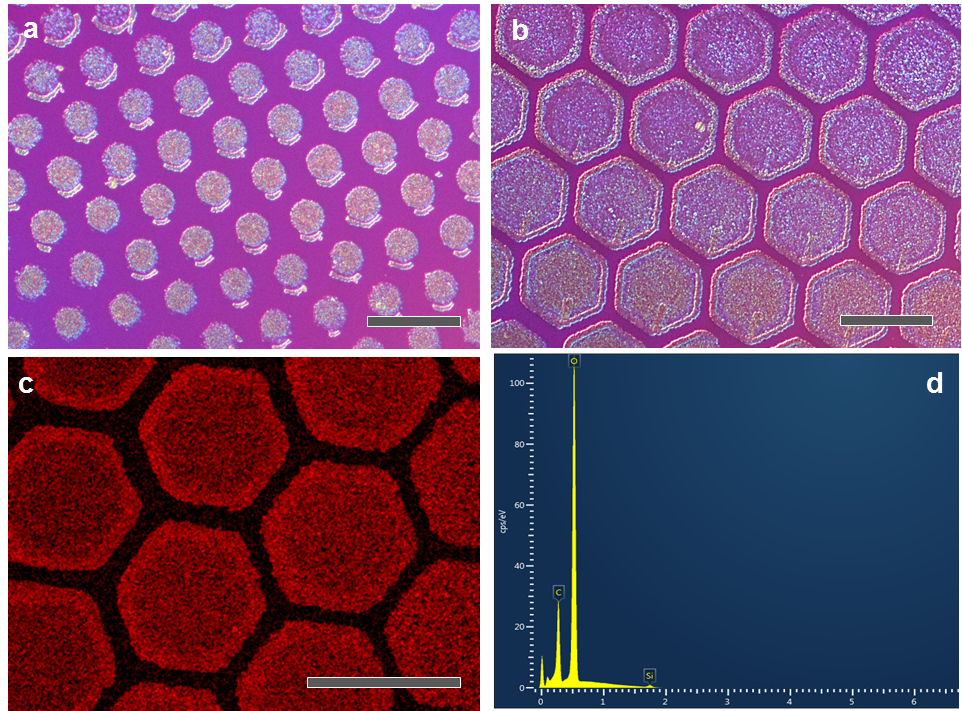
**

**Supplementary Figure 12** | **Micro-patterned PTEB on SiO_2_ wafer**. Optical microscopy image of (**a**) 10 µm and (**b**) 30 µm patterned PTEB on SiO_2_ wafer. (**c**) EDX elemental mapping of carbon and (**d**) EDX spectrum of the PTEB pattern from (**b**) measured at 1.9 kV acceleration voltage. Scale bars for (**a**-**c**): 40 µm.

**
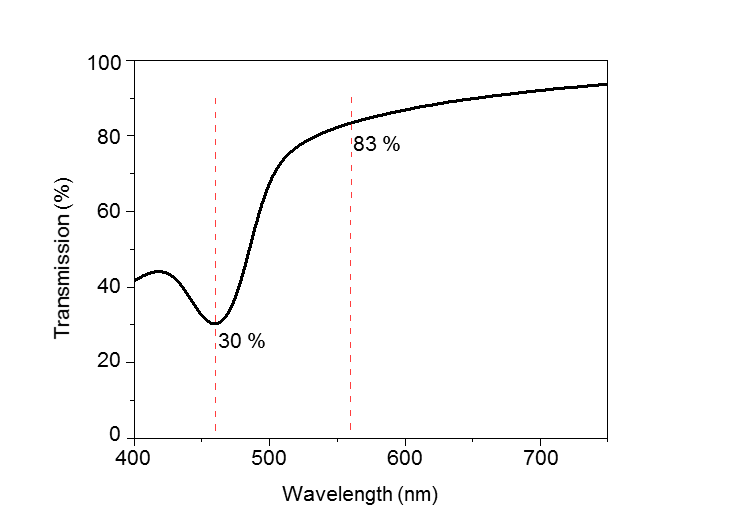
**

**Supplementary Figure 13** | Visible light transmittance spectrum of PTEB films with thickness of *ca.* 210 nm. The films were grown directly on quartz glass and the thickness was determined by AFM.


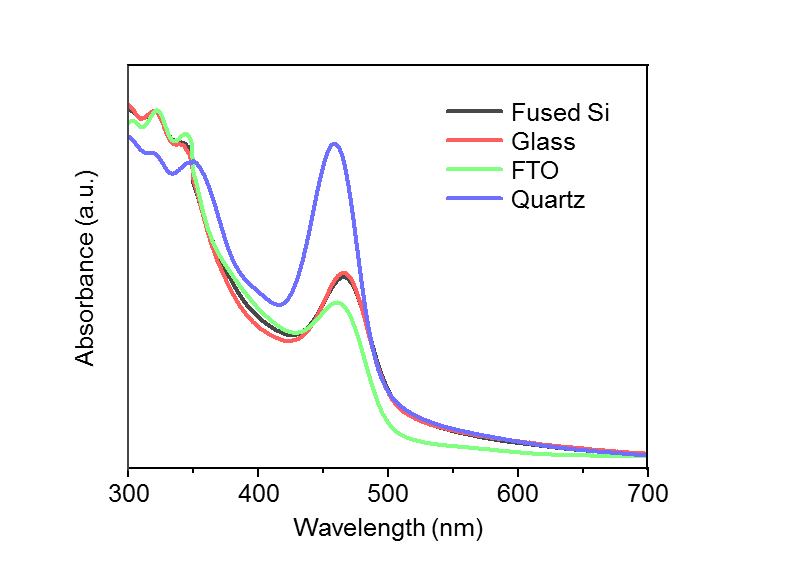


**Supplementary Figure 14** | UV-vis absorption spectra of PTEB films grown on various substrates: fused Si (black curve), glass (red curve), FTO glass (green curve) and quartz (blue curve).


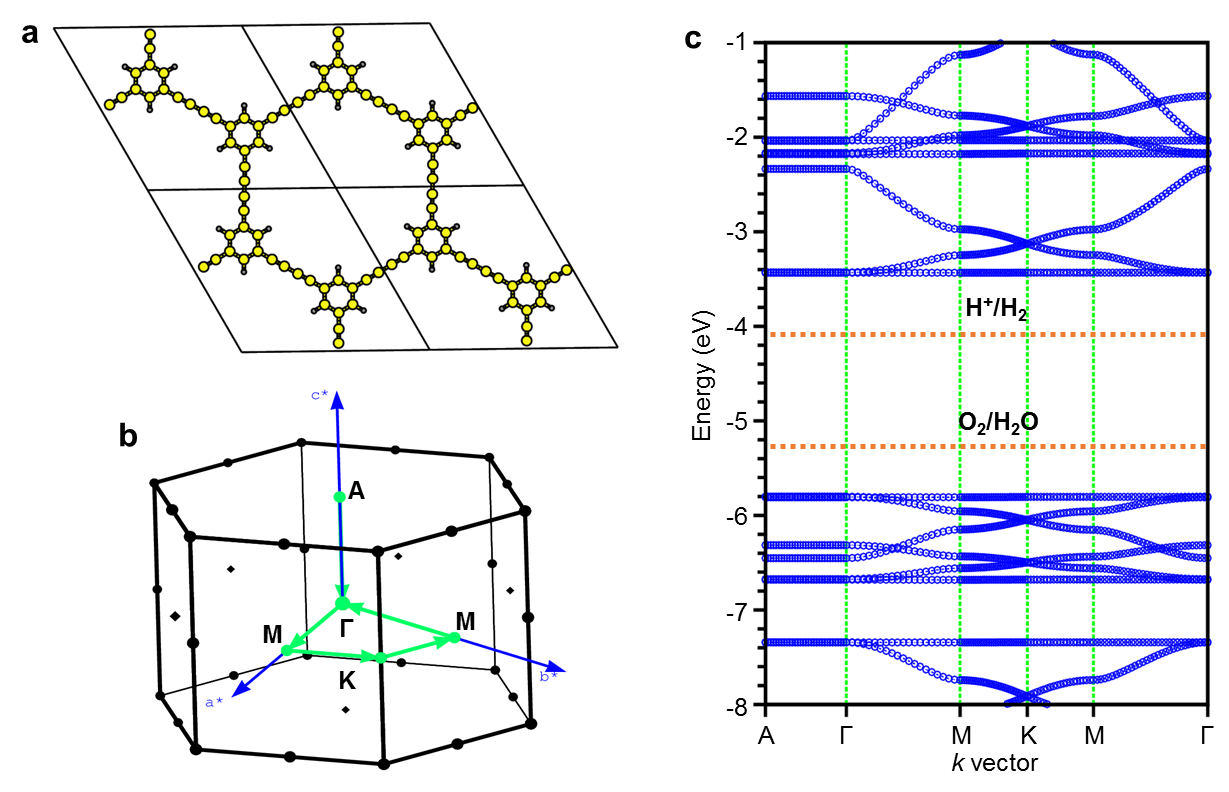


**Supplementary Figure 15** | **DFT calculation of the band structure of PTEB**. (**a**) Atomic structure of simulation supercell for PTEB. (**b**) High symmetry points in Brillouin zone. (**c**) Calculated band structure for PTEB by using vacuum level as reference. All the energy levels are aligned according to vacuum level. The HER potentials (at pH = 7) well located in the band gap of PTEB. The conduction band (CB) and valence band (VB) of PTEB are possible to catalyse HER. The calculated band gap is 2.40 eV, which agrees with the experimental value of 2.51 eV.


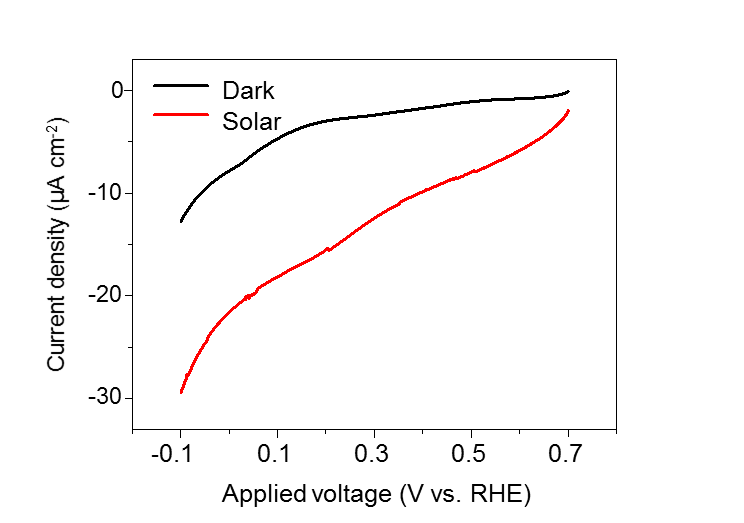


**Supplementary Figure 16** | Linear scanning voltammetry (LSV) curves of PTEB photocathode under dark and simulated sunlight irradiation (100 mW cm^-2^).


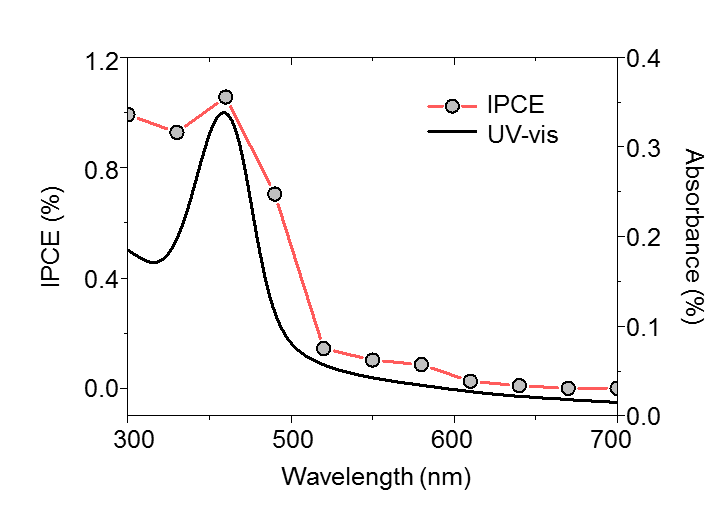


**Supplementary Figure 17** | The incident-photon-to-current-conversion-efficiency (IPCE) spectrum of the PTEB photocathode in 0.01 M in 0.01 M Na_2_SO_4_ under AM 1.5G irradiation. A maximum IPCE value of 1.06% was achieved at 460 nm on PTEB photocathode. Although there is considerable scope for improvement in either photoelectrode layout, co-catalyst, or both^2^, such value is well comparable to those in previous reports of polymeric carbon nitrides^3^, red phosphorus^4^, and even some transition metal dichalcogenides^5^.


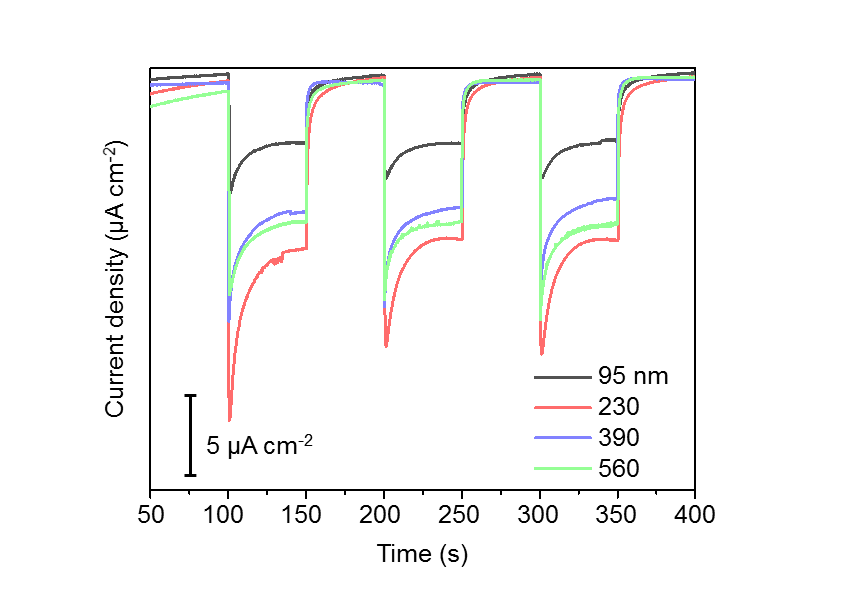


**Supplementary Figure 18** | Transient photocurrent density *vs.* time of PTEB photocathode of various thickness (95, 230, 390 and 560 nm) at a bias of 0.3 V *vs.* RHE (*i.e.* -0.3 V *vs.* Ag/AgCl) under intermittent irradiation in 0.01 M Na_2_SO_4_. A photocurrent density of 4.2 μA cm^-2^ could be obtained on a 95 nm thick PTEB layer at a bias of 0.3 V *vs.* RHE. The value reach to *ca.* 10 μA cm^-2^ when the layer of PTEB nanofibers film increased to *ca.* 230 nm thick, since the light adsorption was improved. However, the photocurrent drops continuously when thickness of PTEB was further increased to 390 nm and 560 nm, respectively, due to their long transport distance (from PTEB surface to electrode).


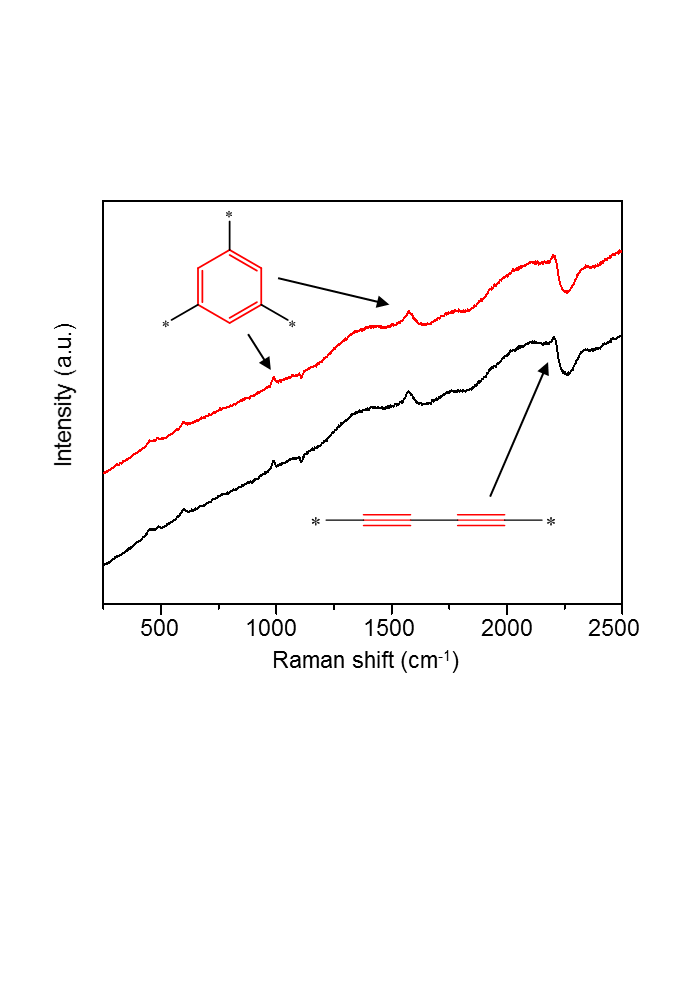


**Supplementary Figure 19** | Raman spectra of PTEB grown on titanium plate before (black curve) and after PEC test (red curve). The Raman line at 2209 cm^-1^ (assigned to the conjugated diacetylenic linkages) and the one at 989 cm^-1^ (assigned to the breathing of the aromatic rings of PTEB) are retained after the PEC measurement, confirming that the PTEB nanofibers are stable under the PEC conditions.


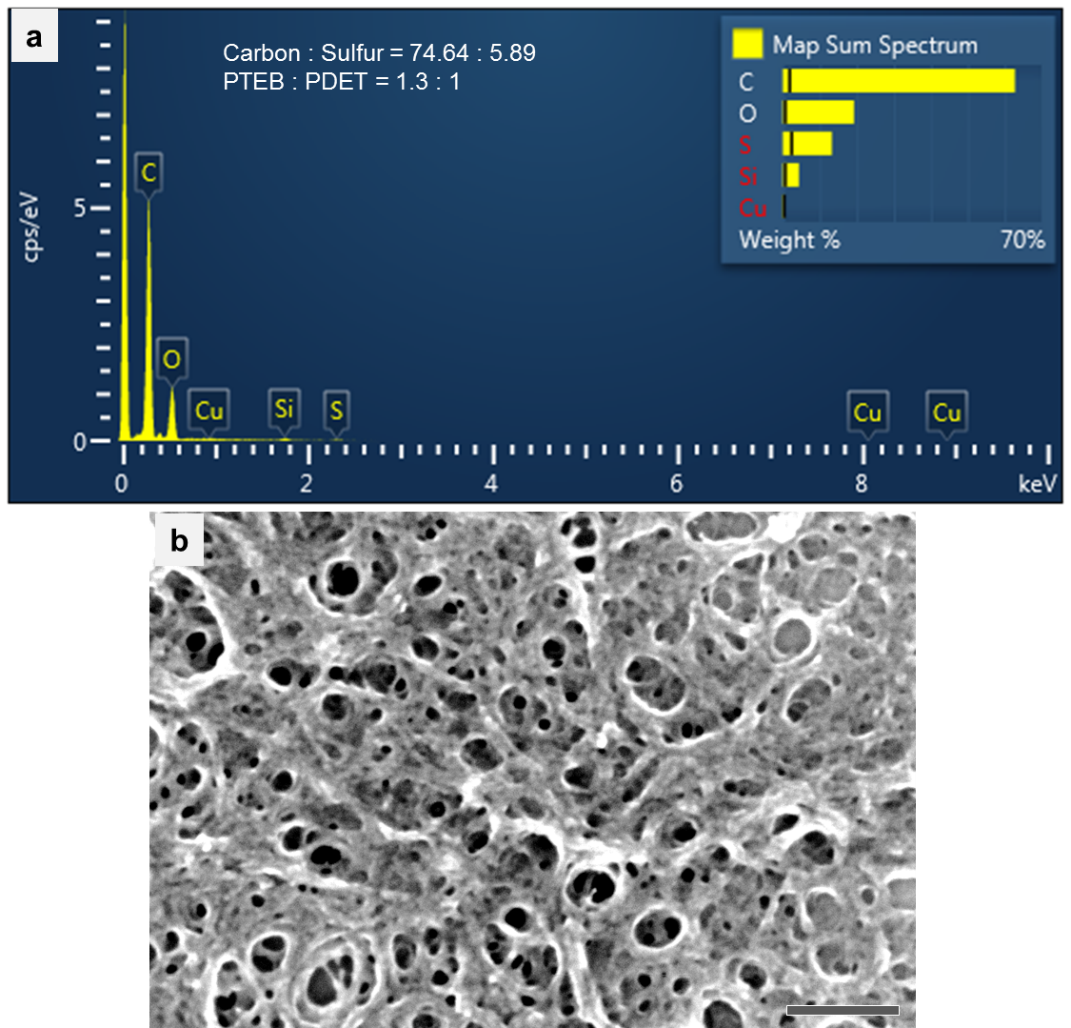


**Supplementary Figure 20** | **EDX spectrum and morphology of PTEB_1.3_-*co*-PDET_1_ copolymer film**. (**a**) EDX spectrum of PTEB_1.3_-*co*-PDET_1_ on SiO_2_/Si wafer measured at 3 kV acceleration voltage. The ratio of PTEB and PDET in the copolymer structure can be estimated by the ratio of carbon and sulphur from the EDX spectrum. Therefore, the copolymer can be more precisely defined as PTEB_1.3_-*co*-PDET_1_. (**b**) SEM image reveals that the PTEB_1.3_-*co*-PDET_1_ copolymer shows larger nanofiber (bundle) morphology than PTEB. Scale bar: 100 nm.


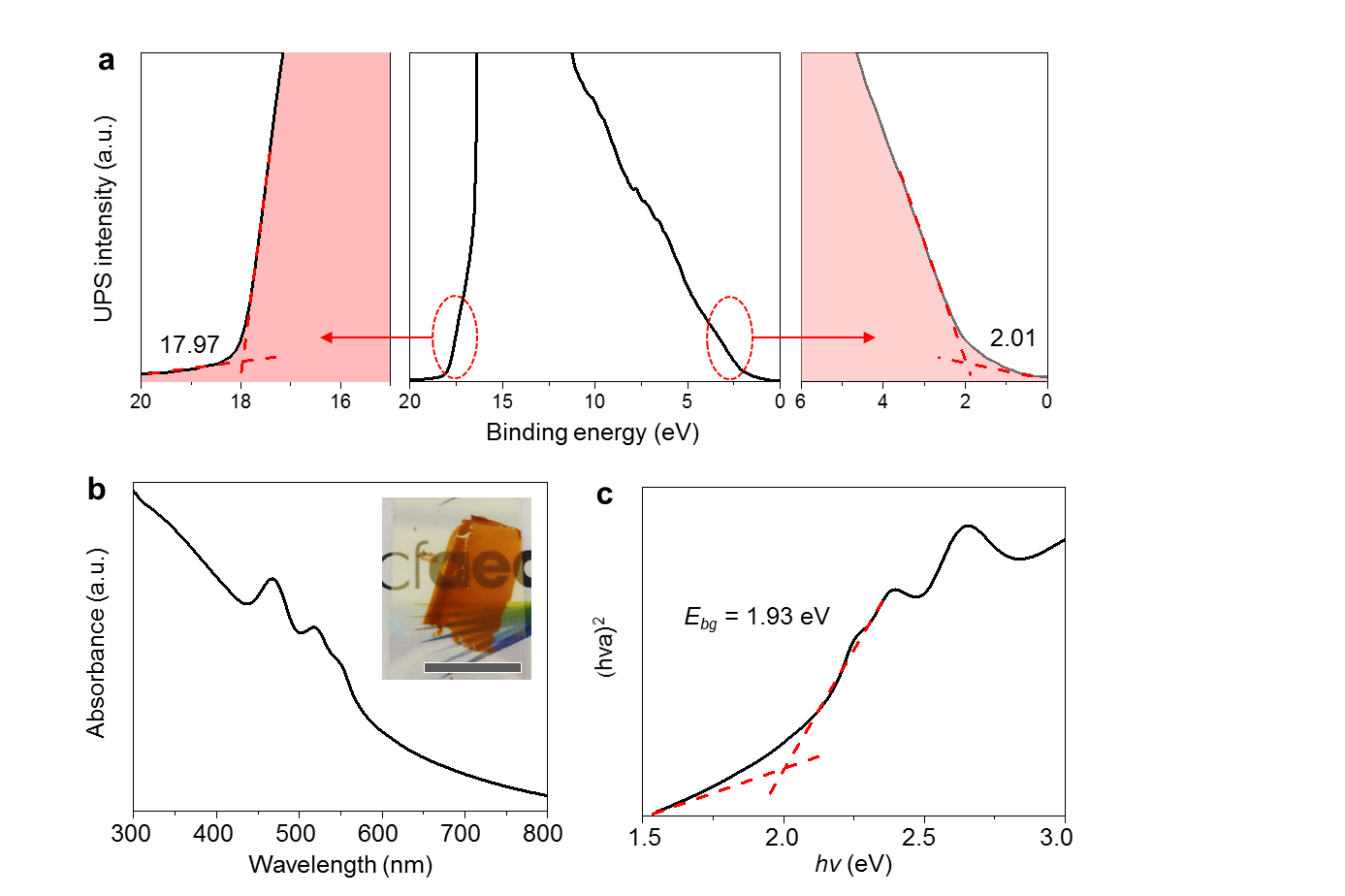


**Supplementary Figure 21 | Optical and electronic structure of PTEB_1.3_-*co*-PDET_1_ copolymer film**. (**a**) UPS spectra (black curve). The dashed red lines mark the baseline and the tangents of the curve. The intersections of the tangents with the baseline give the edges of the UPS spectrum from which the UPS width is determined. (**b**) UV-vis absorption spectra. Inset: digital photograph of the PTEB_1.3_-*co*-PDET_1_ film (*ca.* 230 nm) transferred to a PET substrate; scale bar: 10 mm. (**c**) (*hvα*)^2^ *vs.* *hv* curve (black curve). The horizontal (relatively) dashed red line marks the baseline; the other dashed line marks the tangent of the curve. The value at the intersection is the bandgap: *E_bg_* = 1.93 eV.


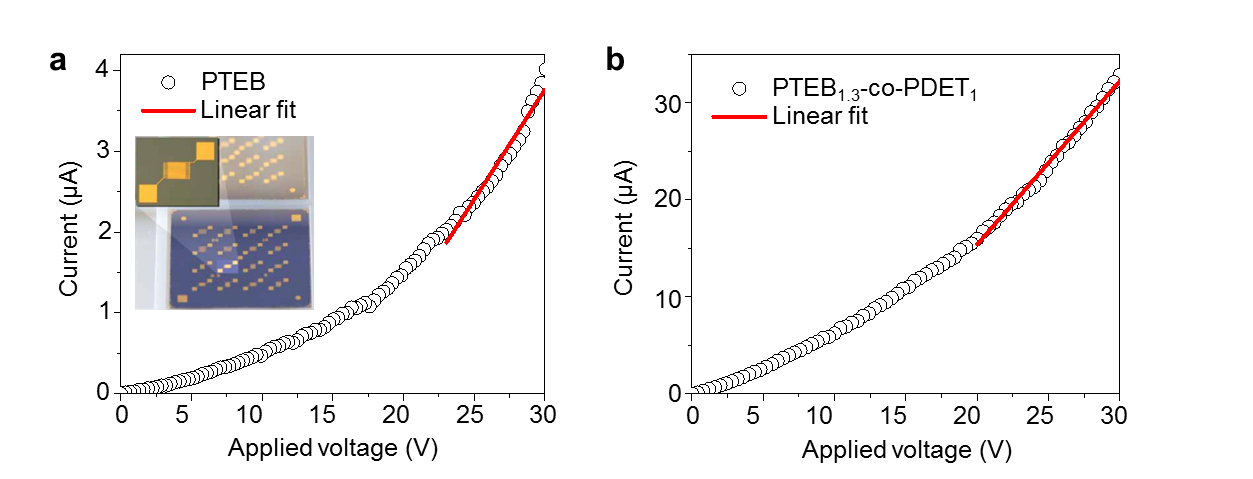


**Supplementary Figure 22** | **Electronic comparison of the PTEB and PTEB_1.3_-*co*-PDET_1_ films**. Representative *I*–*V* characteristic curves of (**a**) PTEB and (**b**) PTEB_1.3_-*co*-PDET_1_ on the 2.5 µm channel OFET device. In both panels, the average thickness of the polymer films is *ca.* 230 nm, and both films exhibit semiconductor-like characteristics^15^. *R* of the sample was estimated from the inverse slope of the *I*-*V* curve. When the *I*-*V* curve is not linear, the slope of the curve was estimated from the linear fit of the curve. Thus, the conductivity, *σ*, of the PTEB_1.3_-*co*-PDET_1_ copolymer film was found to be of 1.9 × 10^-5^ S/cm, and about an order of magnitude greater that of pristine PTEB film (*i.e.* 3.0 × 10^-6^ S/cm).


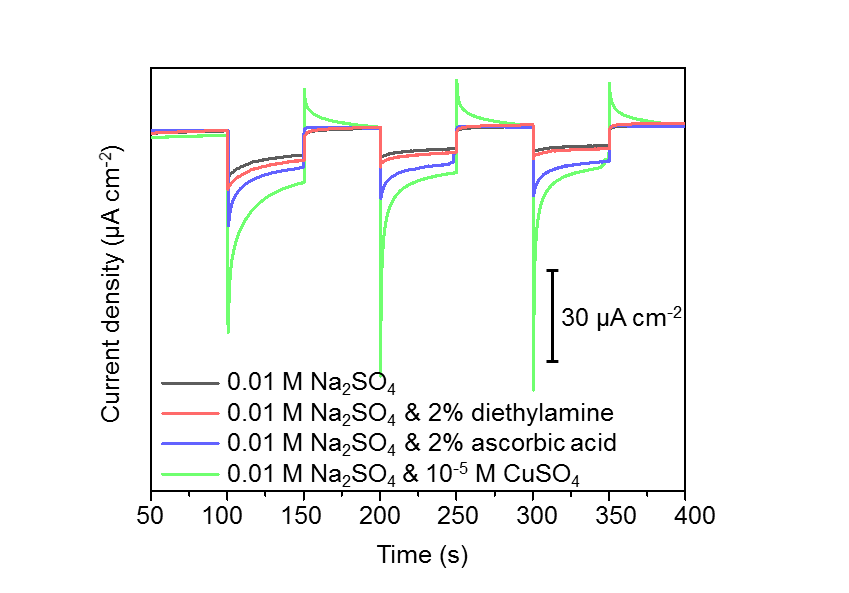


**Supplementary Figure 23** | Transient photocurrent density *vs.* time of PTEB photocathode at a bias of 0.3 V *vs.* RHE (*i.e.* -0.3 V *vs.* Ag/AgCl) under intermittent irradiation in various electrolytes: 0.01 M Na_2_SO_4_ (black curve), hole scavenger diethylamine (2%) in 0.01 M Na_2_SO_4_ (red curve), hole scavenger ascorbic acid (2%) in 0.01 M Na_2_SO_4_ (blue curve), and electron scavenger Cu^2+^ (10^-5^ M) in 0.01 M Na_2_SO_4_ (green curve).


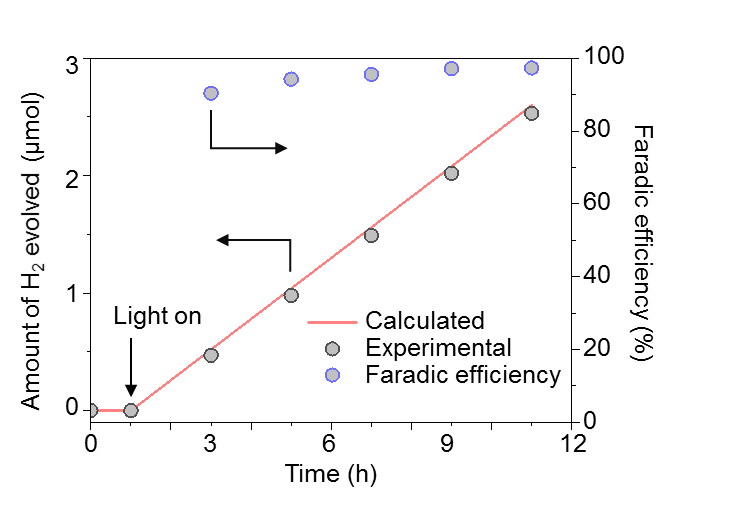


**Supplementary Figure 24** | Amount of evolved H_2_ (black spots), recorded charge carrier (red line) and corresponding faradic efficiency on PTEB electrode in PEC cell with 0 V *vs.* RHE of applied bias in 0.01 M Na_2_SO_4_ under AM 1.5G irradiation (100 mW cm^-2^). The reaction led to 2.53 µmol H_2_ evolution in 10 h reaction on the basis of a PTEB nanofiber film of *ca.* 1 cm^-2^ size and *ca.* 230 nm thick.


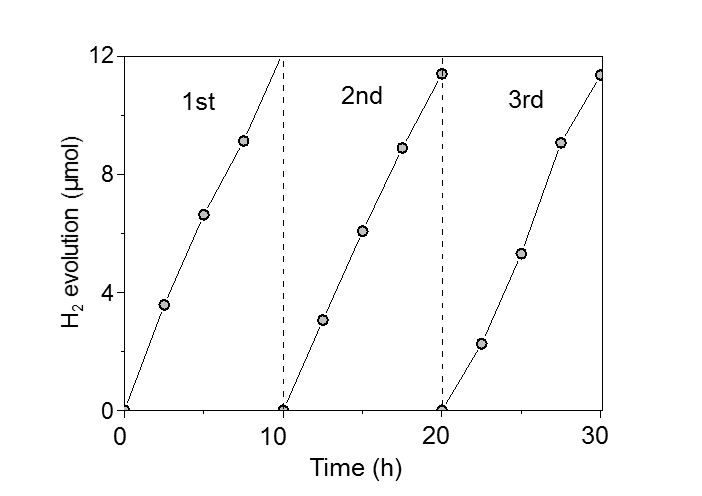


**Supplementary Figure 25** | Cycle runs for the photocatalytic H_2_ production over PTEB nanofibers film (3 × 3 cm^-2^, *ca.* 230 nm thick) under visible light irradiation (λ > 420 nm). The reaction led to 11.4 µmol H_2_ gas in 10 h reaction and without noticeable deterioration of the activity within 30 h.

**
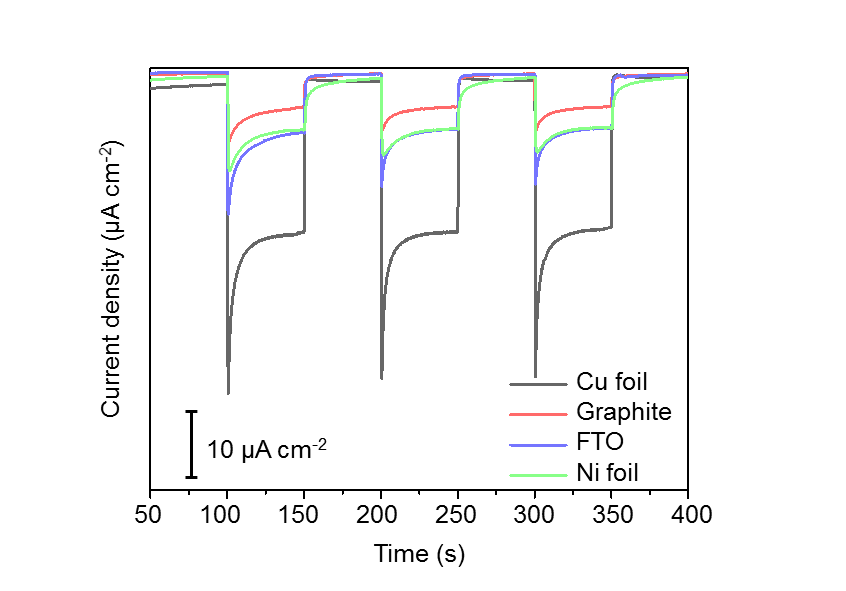
**

**Supplementary Figure 26** | Transient photocurrent density *vs.* time of PTEB photocathode prepared on various conductive substrates (Cu foil, graphite, FTO glass and Ni foil) at a bias of 0.3 V *vs.* RHE under intermittent irradiation in 0.01 M Na_2_SO_4_. The highest photocurrent density was observed on PTEB grown Cu foil with *ca.* 18.6 µA cm^-2^ at 0.3 V *vs.* RHE (*i.e.* -0.3 V *vs.* Ag/AgCl), and lowest one (3.9 µA cm^-2^ at 0.3 V *vs.* RHE) from graphite surface. This is reasonable when the morphologies of the PTEB nanofibers are compared (Fig. 2 and Fig. S11), since the graphite surface only give large bundles (which are not favourable to charge transfer) of PTEB fibres with diameter of *ca.* 40 nm (Fig. 2b), comparing to that of obtained on Cu surface (*ca.* 10 nm, Fig. 1e). The strong effect of size and morphology of the structure on the photocatalytic activity of semiconductors and has been extensively investigated, such as poly(diphenylbutadiyne)^16^, TiO_2_^17^, BiVO_4_^18^. Unfortunately, the Cu substrate may not be suitable as electrode for PEC reaction, due to the possibility of electrochemical corrosion of Cu in the cell.

**Supplementary Table**

**Supplementary Table 1** | **Reported photocurrent densities of metal-free photocathodes in PEC cells.**

| Photocathode | Photocurrent density (J) at the applied potential | Potential* | Electrolyte | Light source |
| --- | --- | --- | --- | --- |
| *g*-C_3_N_4_^6^ | 0.3 µA cm^-2^ | -1.0 V *vs.* Ag/AgCl | 0.5 M Na_2_SO_4_ | 300 W Xenon lamp, λ > 420 nm |
| *g*-C_3_N_4_^7^ | 1.2 µA cm^-2^ | -0.2 V *vs.* Ag/AgCl | 0.1 M KCl | 150 W Xe lamp, λ > 420 nm |
| *g*-C_3_N_4_@C^8^ | *ca.* 0.7 µA cm^-2^ | -0.1 V *vs.* Ag/AgCl | 0.2 M Na_2_SO_4_ | Keithley 6300 semiconductor analyzer |
| *g*-C_3_N_4_-*co*-barbituric acid^9^ | 0.7 µA cm^-2^ | -0.2 V *vs.* Ag/AgCl | 0.2 M Na_2_SO_4_ | 500w xenon lamp, λ > 420 nm |
| C,O-*g*-C_3_N_4_^10^ | *ca.* 0.4 µA cm^-2^ | -0 V *vs.* Ag/AgCl | 0.1 M Na_2_SO_4_ | 300 W xenon arc lamp,  100 mW cm^-2^ |
| Alg-5-CN^11^ | *ca.* 0.4 µA cm^-2^ | -0.2 V *vs.* Ag/AgCl | 0.1 M KCl | A solar simulator, AM 1.5G, *ca.* 84 mW cm^-2^ |
| C_3_N_3_S_3_^12^ | *ca.* 0.005 µA cm^-2^ | -0.2 V *vs.* Ag/AgCl | 0.2 M Na_2_SO_4_ | 300 W Xenon lamp, λ > 420 nm |
| B_4.3_C^13^ | 46 µA cm^-2^ | -0.76 V *vs.* Ag/AgCl | 0.01 M Na_2_SO_4_ | 300 W Xenon lamp, 64 mW cm^-2^ |
| B_13_C_2_^13^ | 16 µA cm^-2^ | -0.76 V *vs.* Ag/AgCl | 0.01 M Na_2_SO_4_ | 300 W Xenon lamp, 64 mW cm^-2^ |
| Red P^14^ | 0.09 µA cm^-2^ | -1.0 V *vs.* Ag/AgCl | N/A | Cole-Parmer illuminator, 41720 series, 200 mW cm^-2^ |
| Bulk-fibrous P^4^ | 0.35 µA cm^-2^ | -0.4 V *vs.* Ag/AgCl | 0.1 M Na_2_SO_4_ | 300 W Xenon lamp, 100 mW cm^-2^ |
| Our work:  PTEB | ***ca.* 10 µA cm^-2^** | -0.3 V *vs.* Ag/AgCl  (*i.e.,* 0.3 V *vs.* RHE) | 0.01 M Na_2_SO_4_ | 200 W Xenon lamp (100 mW cm^-2^), AM 1.5G filter |
| Our work:  PTEB_1.3_-*co*-PDET_1_ | ***ca.* 21 µA cm^-2^** | -0.6 V *vs.* Ag/AgCl (*i.e.,* 0 V *vs.* RHE) | 0.01 M Na_2_SO_4_ | 200 W Xenon lamp (100 mW cm^-2^), AM 1.5G filter |

* The follow equation was used to convert the potential *vs.* Ag/AgCl to RHE potential: *E*_RHE_ = *E*_Ag/AgCl_ + 0.059 pH + *E*_Ag/AgCl_^0^ (*E*_Ag/AgCl_^0^ = 0.199 V); and the potential *vs.* SCE to RHE potential: *E*_RHE_ = *E*_SCE_ + 0.059 pH + *E*_SCE_^0^ (*E*_SCE_^0^ = 0.242 V)

**Supplementary Methods**

The topography of the surfaces, the microstructure of the materials and the morphology of the components of the samples were characterized using a field emission scanning electron microscope (FESEM) (Carl Zeiss Gemini 500) equipped with an energy-dispersive X-ray (EDX) spectrometer. Transmission electron microscopy (TEM) images were obtained using a Cs corrected TEM (Carl Zeiss Libra 200) operated at 200 kV. For TEM studies, the samples were mounted directly on a copper grid. TEM image of the PTEB nanofibers were taken on those grown on the edge of the grid. Optical images were acquired in differential interference mode using an optical microscope (Carl Zeiss AxioScope A1).

Raman spectra were acquired on confocal Raman microscope (NT-MDT) using a 532 nm (2.33 eV) laser. A background-correction was performed on the Raman data in Fig. 1f for a better comparison with simulated result. Fourier transform infrared spectra (FTIR) were collected with a BRUKER TENSOR II spectrometer.

X-ray photoelectron spectroscopy (XPS) and UV photoelectron spectroscopy (UPS) were performed in ultra-high vacuum (base pressure 10^-10^ mbar) with an ESCALAB™ 250Xi XPS Microprobe (Thermo Scientific™), using Al Kα X-Ray source (hυ = 1486.6 eV, monochromatized) and HeI discharge lamp for excitation, respectively. Pass energy was 200 eV for survey XPS spectrum, 20 eV for high-resolution C1s spectrum, and 3 eV for the UPS spectrum.

UV-vis spectroscopy was performed on NR 5000 (Aglient technologies, 172 Germany) using the PTEB film grown on quartz glass in transmission mode.

*I*–*V* curves of PTEB and PTEB_1.3_-*co*-PDET_1_ were measured using a semiconductor analyzer (Keithley 4200) at 25 °C and relative humidity (RH) = 20%. Data were collected over a voltage range of -30 to 30 V using the linear sweep mode. The devices were prepared by transferring PTEB and PTEB_1.3_-*co*-PDET_1_ films, respectively, on commercial OFET substrates (Fraunhofer IPMS, Dresden).

**Supplementary References**

1. Merrick, J. P., Moran, D. & Radom, L. An evaluation of harmonic vibrational frequency scale factors. *J. Phys. Chem. A* **111**, 11683-11700 (2007).

2. Paracchino, A., Laporte, V., Sivula, K., Gratzel, M. & Thimsen, E. Highly active oxide photocathode for photoelectrochemical water reduction. *Nat. Mater.* **10**, 456-461 (2011).

3. Zhang, P., Li, X. H., Shao, C. L. & Liu, Y. C. Hydrothermal synthesis of carbon-rich graphitic carbon nitride nanosheets for photoredox catalysis. *J. Mater. Chem. A* **3**, 3281-3284 (2015).

4. Hu, Z. F., Yuan, L. Y., Liu, Z. F., Shen, Z. R. & Yu, J. C. An elemental phosphorus photocatalyst with a record high hydrogen evolution efficiency. *Angew. Chem. Int. Ed.* **55**, 9579-9584 (2016).

5. Yu, X. Y., Prevot, M. S., Guijarro, N. & Sivula, K. Self-assembled 2D WSe_2_ thin films for photoelectrochemical hydrogen production. *Nat. Commun.* **6**, 7596 (2015).

6. Guo, Y. F. *et al.* A rapid microwave-assisted thermolysis route to highly crystalline carbon nitrides for efficient hydrogen generation. *Angew. Chem. Int. Ed.* **55**, 14693-14697 (2016).

7. Zhang, Y. J., Thomas, A., Antonietti, M. & Wang, X. C. Activation of carbon nitride solids by protonation: morphology changes, enhanced ionic conductivity, and photoconduction experiments. *J. Am. Chem. Soc.* **131**, 50-51 (2009).

8. Han, Q., Wang, B., Gao, J. & Qu, L. T. Graphitic carbon nitride/nitrogen-rich carbon nanofibers: highly efficient photocatalytic hydrogen evolution without cocatalysts. *Angew. Chem. Int. Ed.* **55**, 10849-10853 (2016).

9. Zhang, J. S. *et al.* Synthesis of a carbon nitride structure for visible-light catalysis by copolymerization. *Angew. Chem. Int. Ed.* **49**, 441-444 (2010).

10. Chana, D. K. L. & Yu, J. C. Facile synthesis of carbon- and oxygen-rich graphitic carbon nitride with enhanced visible-light photocatalytic activity. *Catalysis Today*, 10.1016/j.cattod.2017.05.017 (2017).

11. Zhang, Y. J. *et al.* Biopolymer-activated graphitic carbon nitride towards a sustainable photocathode material. *Sci. Rep.* **3**, 2163, (2013).

12. Zhang, Z. Z. *et al.* Organic semiconductor for artificial photosynthesis: water splitting into hydrogen by a bioinspired C_3_N_3_S_3_ polymer under visible light irradiation. *Chem. Sci.* **2**, 1826-1830 (2011).

13. Liu, J. K. *et al.* Boron carbides as efficient, metal-free, visible-light-responsive photocatalysts. *Angew. Chem. Int. Ed.* **52**, 3241-3245 (2013).

14. Wang, F. *et al.* Red phosphorus: an elemental photocatalyst for hydrogen formation from water. *Appl. Catal. B-Environ.* **111**, 409-414 (2012).

15. Khanduyeva, N. *et al.* Grafting of poly (3-hexylthiophene) from poly(4-bromostyrene) films by kumada catalyst-transfer polycondensation: revealing of the composite films structure. *Macromolecules* **41**, 7383-7389 (2008).

16. Ghosh, S. *et al.* Conducting polymer nanostructures for photocatalysis under visible light. *Nat. Mater.* **14**, 505-511 (2015).

17. Prieto-Mahaney, O. O., Murakami, N., Abe, R. & Ohtani, B. Correlation between photocatalytic activities and structural and physical properties of titanium(IV) oxide powders. *Chem. Lett.* **38**, 238-239 (2009).

18. Jeong, S. Y. *et al.* Enhanced photocatalytic performance depending on morphology of bismuth vanadate thin film synthesized by pulsed laser deposition. *ACS Appl. Mater. Inter.* **9**, 505-512 (2017).
